# Immune Specific and Tumor-Dependent mRNA Vaccines for Cancer Immunotherapy: Reprogramming Clinical Translation into Tumor Editing Therapy

**DOI:** 10.3390/pharmaceutics16040455

**Published:** 2024-03-25

**Authors:** Theodora Katopodi, Savvas Petanidis, Eirini Grigoriadou, Doxakis Anestakis, Charalampos Charalampidis, Ioanna Chatziprodromidou, George Floros, Panagiotis Eskitzis, Paul Zarogoulidis, Charilaos Koulouris, Christina Sevva, Konstantinos Papadopoulos, Panagiota Roulia, Stylianos Mantalovas, Marios Dagher, Alexandros Vasileios Karakousis, Nikolaos Varsamis, Konstantinos Vlassopoulos, Vasiliki Theodorou, Chrysi Maria Mystakidou, Nikolaos Iason Katsios, Konstantinos Farmakis, Christoforos Kosmidis

**Affiliations:** 1 Laboratory of Medical Biology and Genetics, Department of Medicine, Aristotle University of Thessaloniki, 54124 Thessaloniki, Greece; katopodi@auth.gr (T.K.); eirinigr90@hotmail.com (E.G.); 2 Department of Pulmonology, I.M. Sechenov First Moscow State Medical University, Moscow 119992, Russia; 3 Department of Anatomy, Medical School, University of Cyprus, Nicosia 1678, Cyprus; anestakis.doxakis@ucy.ac.cy (D.A.); ccharal@med.duth.gr (C.C.); 4 Department of Public Health, Medical School, University of Patra, 26500 Patra, Greece; ioannachatzi@med.upatras.gr; 5 Department of Electrical and Computer Engineering, University of Thessaly, 38334 Volos, Greece; gefloros@e-ce.uth.gr; 6 Department of Obstetrics, University of Western Macedonia, 50100 Kozani, Greece; peskitzis@gmail.com; 7 Third Department of Surgery, “AHEPA” University Hospital, Aristotle University of Thessaloniki, 55236 Thessaloniki, Greece; pzarog@hotmail.com (P.Z.); ckoulour@auth.gr (C.K.); christina.sevva@gmail.com (C.S.); kostaspap1995@hotmail.com (K.P.); steliosmantalobas@yahoo.gr (S.M.); mariosdag@gmail.com (M.D.); alexanderkarakousis@gmail.com (A.V.K.); dr.ckosmidis@gmail.com (C.K.); 8 European Interbalkan Medical Center, 55535 Thessaloniki, Greece; nikvar83@gmail.com; 9 Department of Medicine, Faculty of Health Sciences, Aristotle University of Thessaloniki, 54124 Thessaloniki, Greece; kvlassop@auth.gr (K.V.); baswtheodorou@hotmail.com (V.T.); chryssa2000@gmail.com (C.M.M.); 10 Medical School, Faculty of Health Sciences, University of Ioannina, 45110 Ioannina, Greece; nickkatsios@hotmail.gr; 11 Pediatric Surgery Clinic, General Hospital of Thessaloniki “G. Gennimatas”, Aristotle University of Thessaloniki, 54635 Thessaloniki, Greece; kostafarmakis@yahoo.gr

**Keywords:** mRNA vaccines, reprogramming, immunoediting, immunotherapy

## Abstract

Extensive research into mRNA vaccines for cancer therapy in preclinical and clinical trials has prepared the ground for the quick development of immune-specific mRNA vaccines during the COVID-19 pandemic. Therapeutic cancer vaccines based on mRNA are well tolerated, and are an attractive choice for future cancer immunotherapy. Ideal personalized tumor-dependent mRNA vaccines could stimulate both humoral and cellular immunity by overcoming cancer-induced immune suppression and tumor relapse. The stability, structure, and distribution strategies of mRNA-based vaccines have been improved by technological innovations, and patients with diverse tumor types are now being enrolled in numerous clinical trials investigating mRNA vaccine therapy. Despite the fact that therapeutic mRNA-based cancer vaccines have not yet received clinical approval, early clinical trials with mRNA vaccines as monotherapy and in conjunction with checkpoint inhibitors have shown promising results. In this review, we analyze the most recent clinical developments in mRNA-based cancer vaccines and discuss the optimal platforms for the creation of mRNA vaccines. We also discuss the development of the cancer vaccines’ clinical research, paying particular attention to their clinical use and therapeutic efficacy, which could facilitate the design of mRNA-based vaccines in the near future.

## 1. Introduction

The COVID-19 pandemic has drawn attention to mRNA-based vaccinations on a global scale. Indeed, years of study into mRNA cancer vaccines in preclinical and clinical trials served as the basis for the quick development and production of the COVID-19 vaccine [1,2]. mRNA-based vaccinations have excellent tolerability, are quickly broken down, and do not integrate into the host genome [3]. In addition, mRNA molecules, due to their nature, can be used as vaccines to stimulate both humoral and cell-mediated immunity. Besides that, the manufacturing of mRNA vaccines is quick and affordable [4]. Modern cancer immunotherapies aim to activate the host’s anti-tumor immunity, edit the tumor’s suppressive microenvironment, and eventually lead to a decrease in tumor size and an improvement in patient survival rates [5]. Cancer vaccines are a promising alternative immunotherapeutic treatment with both preventive and curative potential. The immunologic memory of the immune system enables vaccines that target tumor-associated or tumor-specific antigens (TSAs) to precisely attack and kill malignant cells that overexpress the antigens and achieve a sustained therapeutic response [6]. Consequently, cancer vaccines provide targeted, safe, and acceptable treatment in contrast to alternative immunotherapies. Despite the substantial development of clinical trials for cancer vaccinations, the transformation of cancer vaccinations into effective treatments has been problematic for years, mainly due to mRNA instability, immunogenicity, and ineffective in vivo delivery [7].

## 2. mRNA Vaccine Pharmacology

**Figure 1:**
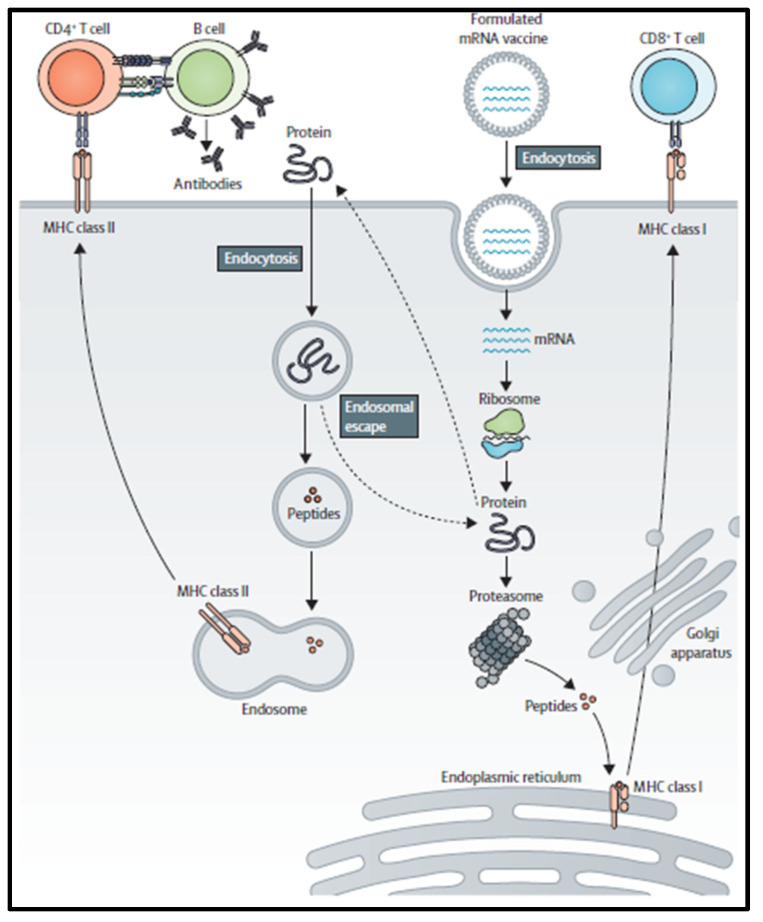
Mode of action of the mRNA-based vaccination. Antigen-presenting cells take up mRNA, and peptides are loaded onto MHC class I for activation of antigen-specific CD8^+^ T lymphocytes. In order to activate CD4^+^ T cells, extracellular proteins or intracellular antigens are either cross-presented on MHC class I or loaded on MHC class II. After receptor-mediated antigen internalization, B cells co-activate CD4^+^ T cells and B-cells whereas protein-specific B cells co-activate CD4^+^ T cells. Reproduced with permission from Ref. [5]. Copyright 2022, Lancet Group.

**Figure 2:**
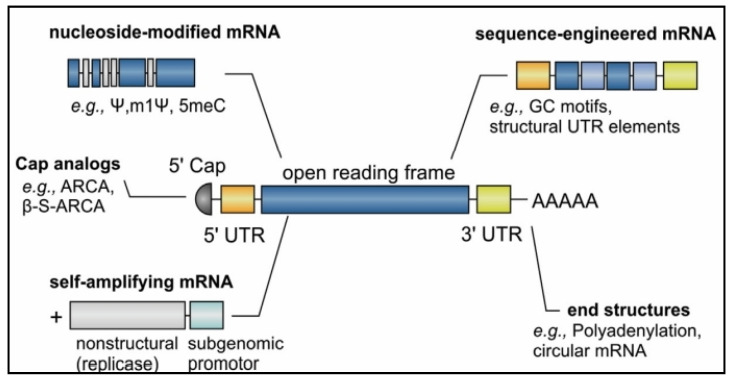
IVT mRNA’s structural characteristics. The structural components of IVT mRNA are described. To alter the stability, translational ability, and immune-stimulatory profile of mRNA, each of these components can be edited and modified. Reproduced with permission from Ref. [13]. Copyright 2021, Elsevier Ltd.

mRNA is a single-stranded macromolecule that is read by ribosomes and translated into proteins in the cytoplasm [8]. It is a single-stranded RNA that corresponds to the genetic sequence of the DNA in cell nuclei. mRNA serves as an intermediary step between the translation of DNA-encoding proteins and the synthesis of proteins by ribosomes in the cytoplasm, [9]. Non-replicating mRNA and virally generated, self-amplifying RNA are the two main forms of RNA that are now being investigated as vaccines. Unlike self-amplifying RNAs, which encode both the antigen and the viral replication machinery that allows for intracellular RNA amplification and abundant protein production, conventional mRNA-based vaccines only encode the antigen of interest and contain 5′ and 3′ untranslated regions (UTRs) [10]. The idea behind using mRNA as a promising cancer vaccine platform (Figure 1) is to introduce the desired transcripts, which encode one or more TSAs, into the cytoplasm of the host cell (the Antigen-presenting cell, APC), where they will be expressed into the desired antigens [2]. Major histocompatibility complexes (MHCs) can then present the expressed TSAs to the surface of APCs to activate anti-tumor immunity. Both antibody driven humoral responses and CD4^+^/CD8^+^ cytotoxic T cell responses, which are valuable for effective clearance of malignant cells, may be induced by mRNA vaccination [11]. Specifically, in vitro-transcribed (IVT) mRNA can be optimally translated and used for therapeutic applications (Figure 2). Using a T7, T3, or Sp6 phage RNA polymerase, IVT mRNA is generated from a linear DNA template. An open reading frame that encodes the target protein, flanking UTRs, a 5′ cap, and a poly(A) tail are the ideal components of the final product [12].

Thus, the mRNA is modified to resemble fully mature mRNA molecules that normally exist in eukaryotic cells’ cytoplasm. Both modified and unmodified non-replicating mRNA and self-amplifying mRNAs (SAMs) have frequently been produced using IVT. This technique uses a linearized DNA template containing the target antigen sequences and a bacteriophage RNA polymerase, such as T3, T7, or SP6 RNA polymerase. mRNA manufacturing is obviously easier, faster, and cleaner than large-scale protein creation and purification since IVT production does not require the use of cells or the regulatory barriers associated with them [14]. After vaccination and cellular uptake by APCs, mRNA is delivered to the cytoplasm, where it undergoes antigen processing and enters the MHC presentation cascade. As a result, APCs display tumor-associated antigens on MHC class I and MHC class II to activate CD8^+^ and CD4^+^ T cells. Additionally, CD4^+^ T cells coactivate B cells that are specific for an antigen and launch a humoral immune reaction [4]. After internalization of extracellular proteins and presentation on MHC class II of B cells, B cells that serve as APCs activate CD4^+^ T cells. After mRNA has been internalized and transported to the cytosol, ribosomes will read it and translate it into proteins in order to produce a properly folded functional protein. The risk of metabolite toxicity will be reduced since the residual IVT mRNA template will be broken down by physiological processes [15]. However, there are a number of restrictions on the use of mRNA in vaccine development. For example, the internalization of naked mRNA by APCs, is not often effective. Lipid nanoparticles, the most often used in vivo RNA delivery vectors, protect messenger RNA from degradation and facilitate endocytosis and endosomal escape. Lipid nanoparticles with positive charges assist in bringing mRNA to negatively charged cells, aiding cytoplasmic endocytosis in the process. In addition, mRNA possesses inherent immunogenicity, which can trigger a pathway connected to interferons and induce innate immunity [16]. Despite the fact that this inherent immunogenicity has an adjuvant-like effect and enhances immune response, it paradoxically promotes mRNA degradation, which lowers antigen expression. Additionally, the contaminants produced during the IVT process, primarily double stranded RNA (dsRNA), will amplify the activation of innate immunity, further restricting mRNA translation. However, recent data from BioNTech report the induction of high T-cell responses after injection of non-modified mRNA in lymph nodes (naked) or intravenous (in liposomes). Specifically, USP18-expressing APCs are insensitive to type I Interferon and still produce proteins to present to the immune system even in the presence of type I interferon.

## 3. Optimization of mRNA Translation

**Figure 3:**
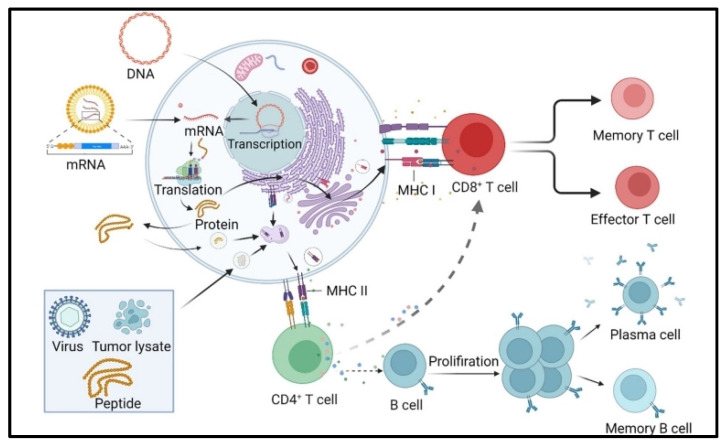
Molecular processing steps of mRNA vaccines. DNA/RNA-based vaccines require additional processing steps than peptide/virus vaccines before being presented to T cells by DCs. Tumor antigens are processed by DCs and transported to the cell surface by MHC I and MHC II molecules. The MHC-peptide complex, the T cell receptor (TCR), and the appropriate receptor-ligand combinations interact to activate T cells. B cells differentiate into plasma cells and memory B cells. Finally, activated T cells differentiate into CD8^+^ effector and memory T cells, which kill tumor cells directly or indirectly. Reproduced with permission from Ref. [21]. Copyright 2022, Springer Nature.

The stability and translation of mRNA are two crucial issues for vaccine development, and they are both significantly influenced by the 5′ and 3′ UTR regions that border the coding sequence (Figure 3) [17]. The half-life and expression of therapeutic mRNAs are significantly extended by these regulatory sequences, which can be obtained from viral or eukaryotic genes. It takes a 5′ cap structure to effectively produce proteins from mRNA molecules. Using a vaccinia virus capping enzyme or by introducing synthetic cap or anti-reverse cap analogues, several forms of 5′ caps can be inserted during or after the transcription reaction [18]. It is necessary to add an appropriate length of poly(A) to mRNA either directly from the encoding DNA template or by utilizing poly(A) polymerase since the poly(A) tail also plays a significant regulatory function in mRNA translation and stability [19]. Additionally, the use of codons affects protein translation. Although the precision of this approach has been questioned, it is a standard practice to replace uncommon codons with regularly occurring synonymous codons that contain an abundance of cognate tRNA in the cytosol. Another method of sequence optimization is enrichment of G:C composition, which raises steady-state mRNA levels in vitro and protein expression in vivo [20].

## 4. Immunogenicity of mRNA Vaccines

The host immune system often triggers an innate immune response by identifying exogenous motifs known as pathogen-associated molecular patterns (PAMPs) using pattern recognition receptors (PRRs) [22]. The primary target cell population of mRNA cancer vaccines is APCs, which exhibit unusually high expression levels of these receptors. IVT mRNA is innately immunostimulatory, as it is recognized by a variety of cell surface receptors [23]. Toll-like receptors (TLR)-7 and -8, which are a type of PRRs, play a major role in the recognition of IVT mRNA inside endosomes. This recognition then activates the MyD88 pathway, which in turn activates the IFN pathway and produces proinflammatory cytokines [4]. Other PRR families, such as retinoic acid-inducible gene-I-like (RIG-I-like) receptors, oligoadenylate synthetase (OAS) receptors, and RNA dependent protein kinase (PKR), sense these exogenous mRNAs in the cytosol [24]. The above PRRs are capable of sensing various RNAs, such as single-stranded RNA (ssRNA) and dsRNA, which prevents the translation of mRNA in the majority of cells. Multiple PRR activation and type I IFN production may paradoxically work in favor of or against anti-cancer treatment [12]. This can be advantageous for vaccination because, in many cases, activation of type I IFN pathways increases antigen presentation, stimulates APC activation, and elicits powerful adaptive immune responses. In addition, free mRNA and exosomes that contain mRNA are being ingested by cells, and these create a background level of mRNA exposure, which has implications for mRNA vaccine efficacy. Specifically, extracellular vesicles (EVs) successfully traverse the cellular membrane and avoid drug delivery barriers such as RNase destruction, endosomal accumulation, phagocytosis, multidrug resistance, cytotoxicity, and immunogenicity due to their biocompatibility with human cells. In addition, RNA-based innate immune sensing may be linked to a reduction in antigen expression, which would attenuate an immune response [25]. Nevertheless, Type I interferon can trigger the development of the immune response, depending on the formulation and site of injection of the mRNA. Particularly during IVT, phage RNA polymerases generate undesired dsRNA that might stimulate innate immunity via PKR, OAS, TLR-3, and MDA-5 (RIG-I-like receptor). The eukaryotic initiation factor (eIF)-2 can be phosphorylated once the PKR is activated, preventing mRNA translation [26]. Additionally, upon binding to OAS, the dsRNA activates RNase L, leading to the destruction of the foreign RNAs. Finally, binding of dsRNA to MDA-5 and TLR-3 can activate Type I IFN, triggering a number of genes that prevent mRNA translation [27].

## 5. mRNA Vaccine Efficacy

**Figure 4:**
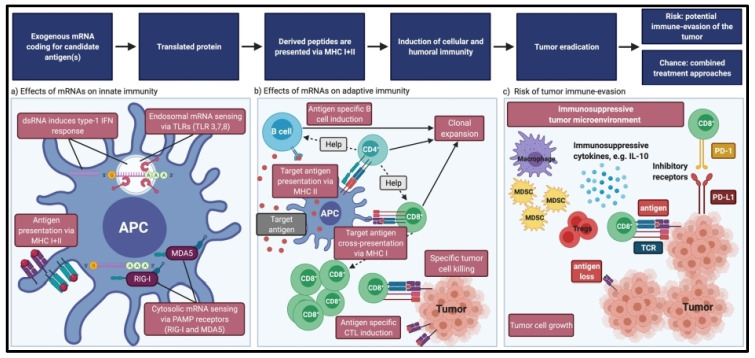
mRNA vaccines’ effects on immunity. (**a**) Exogenous mRNA’s impact on innate immunity. TLRs in the endosomes and receptors like RIG-I and MDA5 in the cytoplasm can both detect exogenous mRNA. Strong IFN1 responses can be triggered by dsRNA. The proteasome will break down the translated protein into peptides, which are then displayed on MHC-I and MHC-II molecules. (**b**) Exogenous mRNA’s impact on adaptive immunity. APCs can cross-present antigens on MHC-I to CD8^+^ T cells and present exogenous antigens on MHC-II to CD4^+^ T cells. B cells and CD8^+^ T cells are assisted by CD4^+^ T cells. Target cell eradication occurs as a result of clonal growth of B and T lymphocytes that are specific for antigens. (**c**) Cancer immune evasion risk. By attracting myeloid-derived suppressor cells (MDSCs), regulatory T cells, and M2 macrophages and producing immunosuppressive cytokines, tumors can produce an immunosuppressive microenvironment. Antigen loss on tumor cells or upregulation of fatigue markers on T cells can both contribute to immune evasion. Reproduced with permission from Ref. [15]. Copyright 2021, Springer Nature.

The inability to respond to treatment directly (primary resistance) or the development of resistance after tumor treatment (secondary resistance) are the two major issues for clinical immunotherapy and trigger tumor escape and subsequent relapse [28,29]. This immunological escape may be facilitated by a variety of underlying immune factors (Figure 4). These factors can be divided into tumor cell “extrinsic” processes, which involve the tumor stromal components, and tumor cell “intrinsic” mechanisms, which are defined by the characteristics of the tumor cell itself [30]. It’s crucial to understand that the same factors that influence the initial resistance to immunotherapy may also influence secondary resistance.

## 6. Tumor Intrinsic Resistance

The downregulation or absence of tumor antigen expression, changes in the antigen processing pathway, and loss of HLA expression are among the tumor-intrinsic factors of resistance to T cell-based immunotherapies, primarily ICI, adoptive cell transfer, and therapeutic vaccination [31]. These factors all prevent T cells from recognizing tumor cells. In fact, the loss of HLA class I expression can result in secondary resistance to the BCG vaccine in bladder cancer, autologous virus-specific T cell transfer in Merkel cell carcinoma, and primary resistance to the therapeutic vaccine (consisting of autologous tumor cells) in melanoma [32]. Immunotherapy-activated T cells’ antitumor effects are impacted by resistance to TNF and IFN signaling. For instance, in patients with melanoma, NSCLC, and bladder cancer, the expression of multiple immune checkpoints by neoantigen-specific CD4^+^ T cells and CD8^+^ T cells was linked to non-responsiveness to the combination of treatment with the neoantigen vaccine and PD1 checkpoint blockade [33].

## 7. Tumor Extrinsic Resistance

**Figure 5:**
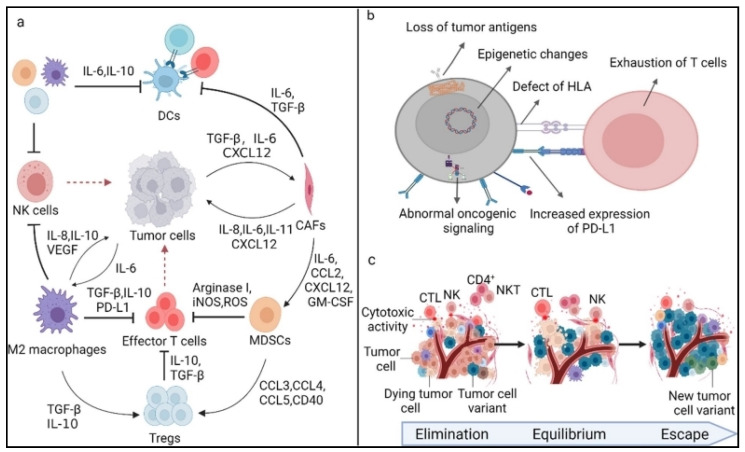
Resistance of cancer vaccines. (**a**) Tumour extrinsic resistance. Immunosuppressive cytokines and immune-suppressive cells, such as CAFs, MDSCs, Tregs, and M2 macrophages, can directly or indirectly prevent the activation of effector T cells and DC-mediated T cells in the TME. (**b**) Tumor intrinsic resistance. Several factors make up a tumor’s intrinsic resistance: mutations in immune-suppressive signaling pathways, loss of tumor antigen expression, changes to antigen processing pathways, loss of HLA expression, epigenetic changes, and increased expression of immunosuppressive ligands. (**c**) From immunosurveillance to tumor escape, immune selection. Reproduced with permission from Ref. [21]. Copyright 2022, Springer Nature.

Resistance to mRNA vaccines may also be caused by ‘extrinsic’ causes [34]. Extrinsic variables associated with immunoresistance include a systemic and local increase in immunosuppressive cells like Tregs, MDSCs, tumor-associated macrophages (TAMs), cancer-related fibroblasts (CAFs), and protumor N2 neutrophils (Figure 5) [35]. These cells, via expressing inhibitory receptors (PD1, CTLA-4), produce immunosuppressive cytokines (IL-10, IL-35, and TGF-β), arginase 1, inducible nitric oxide synthase (iNOS), and ROS. These factors trigger inhibition of systemic and local T cell activation by polarizing local CD4^+^ T cells, neutrophils, and monocytes towards a protumorigenic phenotype [36]. Immunosuppressive cells can also prevent DCs from functioning properly, thus promoting tumor resistance. Specifically, MDSCs are neutrophils and monocytes that have been pathologically activated and exhibit potent immunosuppressive properties [37]. MDSCs are the immunosuppressive barrier that shields tumors from the patient’s immune system and immunotherapy. Nevertheless, the TME’s main core is made up of CAFs. By modifying the extracellular matrix to create thick fibrous stroma, CAFs can reduce DC proliferation and migration, draw MDSCs, and impede T cell invasion [38]. TAMs are divided into pro-tumorigenic M2 and anti-tumorigenic M1 (classically activated) phenotypes [39]. TGF-B1, IL-4, and other Th2 cytokines, as well as immunocomplexes, polarize TAMs into M2 macrophages. Macrophages with the M2 phenotype can activate dormant tumor cells and alter their stromal characteristics to support tumors [40,41]. They may trigger the formation of tumor-related vasculature. In conclusion, there are numerous mechanisms that can underlie cancers’ resistance to mRNA vaccines, necessitating the use of several different strategies to combat it. Depending on the specific resistance mechanism, various treatment approaches may be combined. Improved immunotherapy delivery platforms, better antigen selection, and combination therapy are only a few of the solutions created to address tumor escape and TME immunosuppression [42]. Immunomodulatory compounds, radiotherapy, and chemotherapy drugs could all function in conjunction with mRNA cancer vaccines [43].

## 8. Cell-Based Cancer Vaccines

The tumor cell vaccination strategy is an easy and basic technique that uses allogenic or autologous tumor cells obtained from patients to create cellular vaccines [44]. Tumor cell lines can be genetically altered to increase the immune response against entire tumor cells by adding genes encoding cytokines, chemokines, and co-stimulatory molecules or by suppressing immunosuppressive genes [45]. This method’s drawback is that it can be really challenging to collect enough cells to trigger an efficient immune response. The mechanism of cell-related cancer vaccines is based on DCs, which are highly specialized APCs that stimulate naive T lymphocytes [46]. DCs are loaded with various tumor antigens in the form of DNA, RNA, tumor lysates, tumor-derived proteins, or peptides using several DC-based vaccine development techniques [47]. Different forms of DC vaccinations have been produced recently based on the subpopulation of DCs. Monocyte-derived DCs (Mo-DCs) and leukemia-derived DCs (DCleu) are the two main types of DCs employed in DC vaccine production [48]. DC cancer vaccines have been investigated in phase I, II, and III clinical trials because sufficient numbers of DCs can be collected and cultured [49]. In addition, Sipuleucel-T (Provenge) was the first cell-based vaccine approved by the FDA for hormone-refractory Prostate cancer (HRPC).

## 9. Peptide-Based Cancer Vaccines

To elicit the required immune response, peptide-based cancer vaccines use highly immunogenic tumor-specific peptide antigens [50]. Peptide vaccination strategies are being used to create customized cancer vaccines using synthetic peptides. Antigenic peptides are picked up by APCs and displayed on the cell surface in association with the HLA molecules [51]. The surface antigens are recognized by T cells, which trigger an immune response unique to each tumor type. In comparison to other vaccine types, the peptide-based vaccine method provides a number of benefits, especially in terms of production and safety [52]. For liver and cervical malignancies, respectively, the HBV (Hepatitis B virus) and HPV (Human Papilloma Virus) vaccines are two examples of peptide-based vaccinations. Protamine, a cationic peptide, was employed in numerous early investigations to deliver mRNA vaccines [53]. Through electrostatic contact, protamine naturally condenses mRNA, preventing the encapsulated mRNA from being destroyed by external RNases. The protamine-mRNA complexes can also act as adjuvants by activating TLR-7/8 to cause aTh-1 type immunological response [54]. Protamine-mRNA complexes alone, however, demonstrate weak translation efficiency, which may be caused by the abnormally close contact between protamine and mRNA. In addition, RNA can interact with cationic cell-penetrating peptides (CPPs) [55]. CPPs are thought to assist in the clustering of negatively charged glycosaminoglycans on the cell surface and initiate micropinocytosis, despite the fact that their cell-uptake mechanisms are not entirely known [56]. With positively charged arginine residues on one end and neutral leucine residues on the other, the RALA peptide is an example of an amphipathic arginine-rich CPP. According to studies, RALA-peptide condensed mRNA complexes used for mRNA delivery led to the induction of powerful cytolytic T cell responses following intravenous injection of ex-vivo-loaded DCs [57].

## 10. Nucleic Acid-Based Cancer Vaccines

Nucleic acid vaccines include antigens that are encoded by either DNA or RNA [58]. Nucleic acid vaccines are a potential and alluring vaccine platform because they can provide many antigens with a single vaccination and elicit potent MHC-I-mediated CD8^+^ T cell responses [59]. Nucleic acid vaccines have exhibited benefits over conventional vaccinations, including safety, specificity for eliciting an immune response to the target antigen, elicitation of both humoral and cellular immunological responses, and relative affordability and simplicity of manufacturing [60]. DNAs that have been modified to code for one or more TAs are used in DNA cancer vaccines. DNA vaccines migrate from the cytoplasm to the nucleus after crossing the cell membrane of APCs to begin transcription [61]. The resultant mRNAs move to the cytoplasm, where the host machinery translates them into particular TAs. The APCs are subsequently exposed to the resultant antigens in order to elicit an immunological response [62]. Long-term expression and the poor immunogenicity of DNA vaccines in comparison to other vaccination platforms have made RNA vaccines quite popular [63]. Over the past ten years, preclinical and clinical trials for a number of DNA cancer vaccines have been conducted. mRNA vaccines can carry genetic information encoding TAs in the form of mRNAs, similar to DNA vaccinations. Since mRNA vaccines are translated in the cytoplasm rather than the nucleus, the nucleus is not necessary [64]. Compared to DNA vaccinations, mRNA vaccines have a higher overall immunogenicity. Antigen exposure can be more carefully managed by the transient production of mRNA-encoded antigen, which also lowers the danger of long-term antigen exposure. The RNA vaccine’s drawback is that RNA can be broken down more quickly than DNA [65]. However, there are a number on adjustments that can improve stability. Clinical research of mRNA vaccines has advanced slowly because of issues with stability, the expense of customised manufacturing of patient-specific vaccinations, and delivery [66]. Multiple mRNA vaccines were successfully developed and clinically used in response to the COVID-19 pandemic, demonstrating the platform’s exceptional adaptability, safety, and promise of immunogenicity on a worldwide scale. Different mRNA cancer vaccines are now at various stages of development [67]. In patients with stage III or IV melanoma, the immunostimulant mRNA vaccine TriMix, expressing CD70, CD40L, and a constitutively active version of TLR4, elicited robust CD8^+^ T cell responses and demonstrated improved immune response rates in a phase II clinical trial [68]. Furthermore, Moderna created another immunostimulant mRNA vaccine, mRNA-252, which contains human OX40L, IL-23, and IL-36 and is currently undergoing a clinical trial to treat lymphoma [2].

## 11. Viral-Based Cancer Vaccines

Several viruses possess immunogenic properties by nature, and their genetic makeup can be changed to add TA-encoding genes [69]. Many viruses have served as the foundation for cancer vaccinations. Adenoviruses, poxviruses, and alphaviruses are the most frequently used viral vaccine vectors [70]. The majority of viral vectors are either attenuated or replication-defective varieties. The fact that the immune system reacts to viruses effectively, with both innate and adaptive mechanisms cooperating to induce potent and long-lasting immune responses, is a significant benefit of viral-based vaccines [71]. Vaccines using oncolytic viruses are a cutting-edge and innovative strategy. Oncolytic virotherapy (OVT) is a form of immunotherapy in which oncolytic viruses recognize, attack, and destroy tumor cells while fostering anti-tumor responses. Reactive oxygen species (ROS) and cytokines are produced by tumor cells after infection with the oncolytic virus, which excite immune cells and lead to oncolysis [72]. One such oncolytic viral vaccine is T-VEC, a first-generation recombinant herpes simplex virus product. Due to its simplicity of usage and wide range of host cell tropisms, adenovirus is another employed oncolytic virus in addition to the herpes simplex virus [73]. Finally, the TRICOM vaccine platforms (PROSTVAC and PANVAC) and other viral vector-based vaccines have demonstrated minimal toxicity in a wide range of tumor types, different stages of disease, and in combination with radiation, chemotherapy, and hormone therapy [74]. Preclinical research on TRICOM-based vaccines has shown that they can be utilized to improve vaccine-mediated immune responses and anticancer efficacy in conjunction with radiation, chemotherapy, anti-CTLA4 monoclonal antibodies, and small-molecule targeted treatments [75].

## 12. Lipid mRNA-Based Nanovaccines

In the pharmaceutical sector, lipid nanoparticles (LNPs) have gained attraction as potential delivery systems for a range of medicinal drugs. Moreover, LNPs are now being used in a variety of different industries, including nutrition, medical imaging, cosmetics, and other cutting-edge domains like nanoreactor technology [76]. Living nucleic acids are essential for the efficient protection and delivery of mRNA to cells and are currently in the limelight as an essential part of the COVID-19 mRNA vaccinations. Ionisable lipids, cholesterol, phospholipids, and lipid-linked derivatives of polyethylene glycol are the main components of lipid nanoparticle vaccines [77]. The bilayer structure of the lipid nanoparticle vaccines is supported by cholesterol and phospholipids, which also enhance stability. By preventing the binding of mRNA to plasma proteins, polyethylene glycol extends the time that a nanoparticle is in circulation [78]. Changes in pH levels have an impact on the characteristics of lipid nanoparticle vaccines and make mRNA encapsulation and host-cell endocytosis easier. The first two lipid nanoparticle SARS-CoV-2 vaccines were recently approved, which brought attention to the mRNA nanoparticle vaccine carrier technology [67]. Pembrolizumab is being tested in combination with lipid nanoparticle mRNA cancer vaccines (mRNA-4157) that encode numerous neoantigens as adjuvant therapy in patients with high-risk cutaneous melanoma (NCT03313778 and NCT03897881) [79]. The identical vaccination template (mRNA-4157) was tested both alone and in combination with pembrolizumab in patients with solid tumors that were unable to be surgically removed (NCT03313778). The medication produced neoantigen-specific T cells and had no significant negative side effects. Currently, the lipid nanoparticle-based mRNA cancer vaccine mRNA-5671, which targets four KRAS mutations, is being examined in a phase 1 clinical trial (NCT03948763). Selected patients with KRAS-mutated NSCLC, colorectal cancer, or pancreatic cancer can receive mRNA vaccination alone or in addition to pembrolizumab [80].

## 13. Dendritic Cell mRNA-Based Vaccines

**Figure 6:**
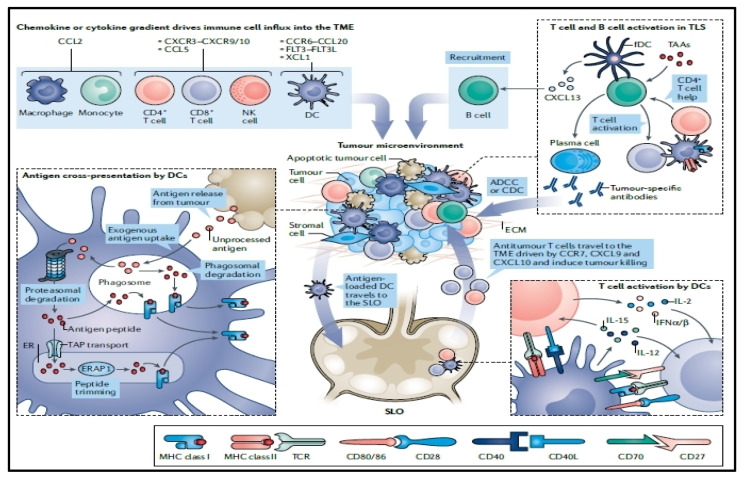
Tumor immunity control. A chemokine gradient directs immune cells into the tumor microenvironment (TME). Dendritic cells (DCs) express tumor antigens on MHC class II or MHC class I molecules (via cross-presentation) within the TME (**bottom left**). The vacuolar pathway or the cytosolic pathway can both be used for cross-presentation. Antigens from endosomes or phagosomes are carried into the cytosol via the cytosolic route, where they are then proteasomally digested and delivered to the endoplasmic reticulum (ER). Peptides are then further modified before being delivered to the cell surface by MHC class I molecules. On the other hand, shorter peptides may be returned to phagosomes after cytosolic proteolytic digestion, loaded onto MHC class I molecules, and then delivered to the cell surface. Antigens are processed and loaded onto MHC class I molecules in phagosomes or endosomes during the vacuolar pathway. In the secondary lymphoid organ (SLO), antigen-loaded DCs move and activate T cells (**bottom right**). Reproduced with permission from Ref. [83]. Copyright 2021, Springer Nature.

Due to their special capacity to initiate immunity and manage or control the type of immune response (Figure 6), DCs have attracted particular attention in immune-treatment methods, making them appealing candidates as carriers of mRNA [81]. The creation of an ex-vivo population of antigen-loaded DCs that can incite powerful and protracted CD8^+^ and CD4^+^ T-cell responses in cancer patients has been the focus of many studies during the last few years [82].

DC-based vaccines with mRNA typically produce weak T-cell responses and exhibit poor clinical effectiveness [45]. However, recent studies indicate that the mRNA-based dendritic vaccines may be able to prevent or postpone disease relapse and can potentially increase overall survival [84]. DC vaccines have been studied in patients with a variety of tumor types in several clinical trials in the last decade, either as a monotherapy or in conjunction with chemotherapy or immunotherapy [85]. In a phase 3 clinical trial, DCs infused with amplified tumor RNA and mRNA-expressing CD40L were administered alongside the tyrosine kinase inhibitor sunitinib to patients with metastatic renal cell carcinoma [86]. The immunization, however, did not increase patient survival. Likewise, DCs loaded with mRNA-expressing tumor-associated antigens were administered to patients with metastatic castration-resistant prostate cancer in a phase 2 trial, but the vaccination did not significantly increase patient survival either [87]. In a phase 2 trial, TriMixDCs expressing TAA-encoding mRNA were administered in combination with the anti-CTLA-4 antibody ipilimumab to patients with metastatic melanoma. 38% of the patients who received treatment responded completely or partially to the combination therapy; however, there was no direct comparison of the outcomes of the two regimens [68]. In a similar manner, DCs loaded with a TAA-encoding mRNA were used to administer vaccinations to remissive patients with acute myeloid leukemia [88]. The 5-year overall survival of the immunized patients was better than that of historical controls, and 43% of patients experienced relapse prevention or delay. Finally, in various phase 1 clinical investigations (NCT00639639, NCT00626483, and NCT02529072), patients with glioblastoma multiforme received DCs loaded with mRNA encoding a CMV (cytomegalovirus) antigen. The elevated expression of CMV proteins in glioblastomas served as the basis for the antigen selection and significantly improved survival compared with the DC control cohort [89].

## 14. Challenges and Future Perspectives

**Figure 7:**
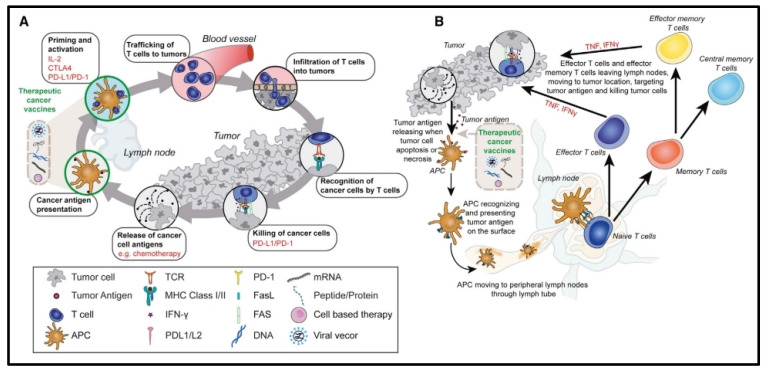
Challenges for the immune mechanisms that induce anti-tumor T cell responses. Therapeutic cancer vaccines provide antigens to dendritic cells that circulate through the lymphatic system and present cancer antigens to naive T cells in an effort to elicit powerful immune responses. Proliferating, multiplying, and moving all across the body, activated cytotoxic T cells are capable of storing long-lasting immunologic memories. (**A**) The effects of vaccine activity and combination immunotherapy on particular stages of the cancer immune cycle. (**B**) Specific to vaccine treatment, T cell activation, effector function, and immunological memory. Reproduced with permission from Ref. [96]. Copyright 2021, Cell Press.

The number of therapeutic mRNA cancer vaccines in clinical trials is increasing quickly as a result of recent scientific breakthroughs that have optimized mRNA transport, administration methods, and increased translational effectiveness [90]. However, several obstacles exist for mRNA vaccine immunogenicity and efficacy, despite significant advancement. For example, the half-life of the mRNA is short and it remains in human tissues for just a few days. The immune response elicits the production of antibodies, which allows the body to develop a certain degree of immunity against the specific pathogen [91]. With the mRNA vaccines, the body won’t have to experience real exposure to the pathogen while still generating an immune response, which would be produced by naturally contracting pathogens. The capacity to pinpoint specific cancer neoantigens is thus one of the most significant developments in therapeutic clinical cancer vaccines. It is still difficult to determine tumor-specific mutations or non-conforming sequences and anticipate the relevant neoepitopes for certain HLA alleles [92]. Future challenges that we need to overcome include the technological and regulatory issues of large-scale production and the good manufacturing practices of customised mRNA vaccines [93]. Validating the vaccine administration methods that are the most practical presents another difficulty. The mode of delivery affects mRNA dispersion and vaccination effectiveness [94]. Although local APCs may easily process intradermally and subcutaneously administered mRNA, these administrations frequently result in significant local injection-site responses. When administered as monotherapy, the majority of mRNA-based cancer vaccines are therapeutic rather than preventive and require many administrations and high vaccination potency to cause a tumor response (Figure 7) [95]. For patients with early-stage cancer or in an adjuvant setting, monotherapy mRNA-based vaccines may be an effective treatment, but it seems unlikely that the vaccines will be successful in treating advanced cancers due to difficulties with the highly immunosuppressive tumor microenvironment in this setting.

When used in conjunction with other immunotherapeutic therapies such as immune checkpoint inhibitors, oncolytic viruses, and adoptive cell therapy, therapeutic mRNA cancer vaccines are more likely to be effective. For example, in comparison to DC-based mRNA vaccines, IVT mRNA antitumor vaccines make up a small portion of melanoma immunotherapeutics in clinical trials, but the encouraging outcomes of IVT mRNA vaccines from preclinical studies indicate their great potential for use as immunotherapies for treating melanoma. In a phase 1 clinical trial, patients with stage III or stage IV melanoma who had a stable illness and a complete response to prior treatment received intranodal injections of non-formulated (naked) mRNA vaccines. With ten carefully chosen neoepitopes, this neoepitope-targeting vaccine encodes a distinct and personalized tumor mutation signature for each patient [5]. Nearly 40% of the patients with stage IV melanoma had vaccine-related clinical responses, and all patients generated T-cell responses against a variety of vaccine-encoded neoepitopes. In addition, patients with resected melanoma (stages IIc, III, and IV) received an intranodally injected, non-formulated mRNA vaccine (ECI-006) in a recently completed phase 1 clinical trial (NCT03394937). The vaccination contained mRNAs for five tumor-associated antigens and three dendritic cell activating compounds (TriMix) [97]. Indeed, across cancer diagnoses, patients receiving these combinations have favorable clinical therapy outcomes. A low-toxic mRNA cancer vaccine is ideal for immunotherapy co-treatment approaches since there is a need for innovative therapy combinations that improve response rates and progression-free survival without producing severe side effects [3]. Checkpoint inhibitors and mRNA vaccines have already been used in several trials. Moderna has recently added a novel checkpoint-targeting cancer vaccine, mRNA-4359, to their vaccine development program. Patients with NSCLC and advanced or metastatic cutaneous melanoma will get the indoleamine 2,3-dioxygenase and PD-L1 antigen-encoding mRNA vaccination. For patients with various cancer diagnoses, BioNTech also combines cemiplimab with the mRNA-based FixVac platform (NCT04526899, NCT04382898, and NCT05142189). BioNTech is also preparing a phase 1/2 trial for a first-line therapy for advanced NSCLC alongside Regeneron.

## 15. Conclusions

A significant achievement in the management of solid tumors is the creation of mRNA cancer vaccines. In this review, we have analyzed the operating principles, improvement techniques, and clinical development of cancer vaccines, which could assist in the development of personalized cancer vaccines in the near future (Table 1). We also emphasized the obstacles to the cancer vaccine’s widespread use, such as tumor resistance, and analyzed the current combination strategies in order to enhance its clinical efficacy.

Nowadays, personalized cancer vaccines can be created quickly with improved immunological efficacy due to the advancement of sequencing technology. A tumor-specific T cell response can be produced by personalized neoantigens with a low level of central immunological tolerance. However, removing tumor cells that express a particular neoantigen causes tumor cell proliferation and chemoresistance. One approach to lessening immune evasion and successfully removing malignancies is to target many neoantigens in a single vaccine. Only a few selected neoantigens may now trigger effective anti-tumorimmune responses; hence, highly effective neoantigens need to be found. Additionally, individuals with advanced tumors that have failed conventional treatment approaches comprise the majority of the therapeutic trial subjects for cancer vaccines. Theoretically, patients with a full immune system, a smaller tumor load, and a higher risk of recurrence are better candidates for cancer vaccine therapy. Therefore, the function of the patient’s immune system and tumor load should be thoroughly taken into account in future clinical studies of cancer vaccines. We believe cancer vaccines are promising immune-therapeutics for establishing immune surveillance and boosting the immune system’s capacity to eradicate tumors. To make cancer vaccines an effective immunotherapy tool, however, much work needs to be performed on finding neoantigens, creating combination therapies, and improving vaccination platforms. Numerous clinical trials need to be conducted to assess the combination of mRNA vaccinations with either cytokine therapy or checkpoint inhibitor therapies in an effort to increase the effectiveness of mRNA anticancer vaccines. mRNA is a potent and adaptable cancer vaccination platform, however, our ability to fight cancer will be significantly strengthened if it is successfully developed toward clinical translation. Precision cancer therapy can also be expanded due to personalized mRNA vaccine platforms. Next-generation sequencing (NGS) technology can be used to create personalized mRNA cancer vaccines that code for particular tumor antigens. Neoantigens and their presentation by the human leukocyte antigen (HLA) can be predicted computationally using a variety of methods. In the past, we have seen how to apply computational methods to rational vaccine design and epitope prediction. Overall, the likelihood of creating successful mRNA vaccines against several tumors is growing as a result of the increasing number of studies and clinical trials of tailored cancer vaccines. 

## Figures and Tables

**Table 1:** Current clinical trial studies employing cancer vaccines for tumor therapy.

NCT Number	Tumor Type/Target	Phase	Category	Status
NCT03190265	Pancreatic Cancer	II	Tumor cell	Recruiting
NCT02451982	Pancreatic Cancer	I/II	Tumor cell	Recruiting
NCT03767582	Advanced PDAC	I/II	Tumor cell	Recruiting
NCT03376477	Multiple Myeloma	II	Tumor cell	Recruiting
NCT03096093	Neoplasms	I/II	Allogeneic cell	Recruiting
NCT03970746	NSCLC	I/II	DC	Recruiting
NCT03059485	AML	II	DC	Recruiting
NCT04523688	Glioblastoma	II	DC	Not yet recruiting
NCT03136406	Pancreatic Cancer/Mutant KRAS	I/II	Virus vector	Active, not recruiting
NCT03632941	Breast Cancer/HER2	II	Virus vector	Recruiting
NCT03547999	Metastatic Colorectal Cancer/MVA-BN-CV301	II	Virus vector	Active, not recruiting
NCT04747002	Acute Myeloid Leukemia/DSP-7888	II	Peptide	Recruiting
NCT04263051	Advanced NSCLC/UCPVax	II	Peptide	Recruiting
NCT03149003	Glioblastoma/DSP-7888	III	Peptide	Recruiting
NCT04206254	Liver Cancer/gp96	II/III	Peptide	Not yet recruiting
NCT04274153	Human Papilloma Virus/Gardasil9	IV	Protein	Recruiting
NCT04090528	Prostate Cancer, pTVG-HP, pTVG-AR	II	DNA	Recruiting
NCT03721978,	Cervical cancer/VGX-3100	III	DNA	Recruiting
NCT04526899	Melanoma Stage III-IV/NY-ESO-1, MAGE-A3, Tyrosinase, and TPTE	II	mRNA	Recruiting
NCT04163094	Ovarian Cancer W-ova1	I	mRNA	Recruiting
NCT03394937	Resected melanoma (stages IIc, III, and IV)/CD40L, CD70, caTLR4, gp100, MAGE-A3, MAGE-C2, and PRAME	I	mRNA	Recruiting
NCT02410733	Melanoma/1 NY-ESO-1, tyrosinase, MAGE-A3, and TPTE	I	mRNA	Recruiting

## Abbreviations

TSA: tumor-specific antigens; UTR: untranslated region; MHC: Major histocompatibility complexes; APC: Antigen-presenting cell; (IVT) mRNA: in vitro transcribed mRNA; SAM: self-amplifying mRNAs; PAMP: pathogen-associated molecular pattern; PRR: pattern recognition receptor; TLR: Toll-like receptor; EV: extracellular vesicle; PKR: protein kinase R; OAS: Oligoadenylate Synthetase; MDA-5: Melanoma differentiation associated gene-5; eIF-2: eukaryotic initiation factor 2; ICI: immune chesk point inhibitor; NSCLC: non-small cell lung cancer; MDSC: myeloid-derived suppressor cell; TAM: tumor-associated macrophage; CAF: cancer-related fibroblast; CTLA-4:Cytotoxic T-lymphocyte associated protein 4; PD-1: programmed cell death protein 1; TME: tumor microenvironment; DC: dendritic cell; HBV: Hepatitis B virus; HPV: human papilloma virus; CPP: cell-penetrating peptide; TA: tumor antigen; OVT: Oncolytic virotherapy; LNPs: lipid nanoparticles; TAA: tumor associated antigen; CMV: cytomegalovirus; CAR: chimeric antigen receptor; CRISPR: clustered regularly interspaced palindromic repeats; Cas9: CRISPR associated protein 9.
